# Neighborhood social organization exposures and racial/ethnic disparities in hypertension risk in Los Angeles

**DOI:** 10.1371/journal.pone.0282648

**Published:** 2023-03-06

**Authors:** Gregory Sharp, Richard M. Carpiano

**Affiliations:** 1 Department of Sociology, Dartmouth College, Hanover, New Hampshire, United States of America; 2 School of Public Policy, University of California, Riverside, Riverside, California, United States of America; New York University Grossman School of Medicine, UNITED STATES

## Abstract

Despite a growing evidence base documenting associations between neighborhood characteristics and the risk of developing high blood pressure, little work has established the role played by neighborhood social organization exposures in racial/ethnic disparities in hypertension risk. There is also ambiguity around prior estimates of neighborhood effects on hypertension prevalence, given the lack of attention paid to individuals’ exposures to both residential and nonresidential spaces. This study contributes to the neighborhoods and hypertension literature by using novel longitudinal data from the Los Angeles Family and Neighborhood Survey to construct exposure-weighted measures of neighborhood social organization characteristics—organizational participation and collective efficacy—and examine their associations with hypertension risk, as well as their relative contributions to racial/ethnic differences in hypertension. We also assess whether the hypertension effects of neighborhood social organization vary across our sample of Black, Latino, and White adults. Results from random effects logistic regression models indicate that adults living in neighborhoods where people are highly active in informal and formal organizations have a lower probability of being hypertensive. This protective effect of exposure to neighborhood organizational participation is also significantly stronger for Black adults than Latino and White adults, such that, at high levels of neighborhood organizational participation, the observed Black-White and Black-Latino hypertension differences are substantially reduced to nonsignificance. Nonlinear decomposition results also indicate that almost one-fifth of the Black-White hypertension gap can be explained by differential exposures to neighborhood social organization.

## Introduction

A prevailing feature across North American and European countries is that ethnic minority adults continue to have significantly higher rates of hypertension than their White adult counterparts [1, 2]. In the United States, recent estimates indicate that Black adults not only have a higher age-adjusted hypertension prevalence than White and Latino adults, but they also have lower rates of high blood pressure treatment and control [2–5]. Racial disparities are even wider when focusing on Los Angeles County, the most populous county in the U.S., where Black hypertension prevalence is more than twice that of White and Latino populations [6, 7]. These racially uneven patterns of hypertension are particularly alarming when considering that high blood pressure continues to be a prominent risk factor for stroke, heart failure, coronary heart disease, and all-cause mortality in the U.S. [2]. And while the contributions of individual risk factors, such as health behaviors [8], socioeconomic status (SES) [9], and psychosocial stressors [10–12] to racial differences in hypertension are well documented, little work has examined how differential exposures to neighborhood social conditions contribute to these inequalities. A better understanding of these community-wide social processes can help inform public health policies and interventions geared toward mitigating the risks of developing high blood pressure, particularly for communities of color in urban areas.

To date, research has found associations between neighborhood structural factors and high blood pressure independent of individual-level characteristics. Specifically, adults are at a heightened risk of having or developing hypertension when living in neighborhoods that are socioeconomically disadvantaged [13–15] or residentially segregated [16, 17], and devoid of healthy lifestyle resources (e.g., healthy food availability, recreational opportunities) within the built environment [18, 19]. There is also evidence, however, that sharing neighborhoods with people of similar racial/ethnic backgrounds (i.e., co-ethnics), particularly for Latinos, is protective of poor health, presumably through the diffusion of healthy behaviors, norms, and information [20, 21]. Additional research has demonstrated that chronic exposures to neighborhood stressors, such as crime and disorder [22–25] and residential segregation [17, 26], explain portions of the racial/ethnic gap in hypertension risk.

Despite prior work on neighborhood stressors, scant evidence exists on the role of neighborhood social organization and hypertension risk and disparities. In particular, collective efficacy—the extent to which neighborhood residents trust and support one another and are willing to intervene on behalf of the collective good [27]—has been linked to health and well-being, including self-rated health [28], obesity risk [29, 30], asthma [31], health behaviors [32, 33], and mental health [34]. The neighborhood social cohesion and informal social control that embody collective efficacy may be associated with hypertension risk through such mechanisms as proliferating pro-health social norms and behaviors, attracting local resources that facilitate physical activity, and ameliorating fears of crime and disorder. A related but distinct construct, neighborhood organizational participation may also associate with better health by connecting residents to integral health-promoting resources both inside and outside the local neighborhood, and by fostering a sense of community that could benefit even those who do not affiliate with organizations [32, 35–37].

There is also a lack of research investigating whether the hypertension effects of neighborhood social organization vary across racial and ethnic populations. And given that low-income, minority-concentrated neighborhoods have the capacity to build collective efficacy and mobilize resources that buffer stressful contextual conditions [35, 38], Black and Latino adults may especially benefit from living in and being exposed to tight-knit, organized communities in Los Angeles. Indeed, some qualitative studies on African Americans, for example, document healthier behaviors (e.g., dietary intake) [32] and outcomes (obesity, high blood pressure) [39, 40] in high collective efficacy communities. Taken together, we hypothesize that exposure to higher levels of neighborhood organizational participation and collective efficacy will be associated with a lower likelihood of being hypertensive and will contribute to racial/ethnic disparities in hypertension. We further contend that living in neighborhoods with greater social organization will matter more for Black and Latino adults than White adults.

Another limitation of existing studies is an overreliance on the residential neighborhood as the only consequential space for hypertension risk. For instance, people tend to spend much of their time outside of their local neighborhood performing routine activities, such as working, shopping, and exercising [41], and daily exposure to these various activity spaces could have consequences for triggering stress and developing high blood pressure. Yet, existing hypertension research conceptualizes neighborhoods as only encompassing the residential context and does not consider the amount of time spent in individuals’ nonresidential spaces. This may be particularly salient in Los Angeles where compared with White individuals, African Americans and Latinos are more likely to live in socioeconomically disadvantaged areas, as well as conduct their daily activities in disadvantaged, under-resourced, and racially isolated neighborhoods [42, 43]. Studies also show that daily mobility is facilitated or restricted by adults’ race/ethnicity, SES, and the characteristics of their activity spaces, which further conditions the duration of exposure to home and away neighborhoods [44]. As a result, not accounting for nonresidential exposures may be a source of confounding that leads to misestimated or biased residential effects on health [45, 46].

To address these research gaps, we use novel longitudinal data from the Los Angeles Family and Neighborhood Survey (L.A.FANS) to examine the role of neighborhood social organizational exposures in adults’ hypertension risk. Our study extends prior work on neighborhoods and hypertension in three important ways. First, we assess social organizational stress-buffering mechanisms (organizational participation, collective efficacy) underexplored in hypertension studies. Second, we employ a counterfactual decomposition technique to estimate the relative contributions of these neighborhood exposure characteristics to racial/ethnic hypertension gaps. Our results indicate that neighborhood social organization is not only associated with a lower risk of being hypertensive, but also accounts for roughly one-fifth of the racial disparity. Finally, compared with studies relying solely on the residential neighborhood, our measures of neighborhood context are more complete by adjusting for the amount of time people spend in their residential neighborhoods, in addition to the neighborhoods in which they conduct their routine activities (i.e., activity spaces).

## Methods

### Data sources

This paper uses longitudinal data from the Los Angeles Family and Neighborhood Survey (L.A.FANS). Administered in two waves (Wave 1 in 2000–2002 and Wave 2 in 2006–2008), L.A.FANS is a stratified random sample of 65 census tracts in Los Angeles County, California sampled from three tract poverty strata: very poor (tracts in the 90th or above percentile); poor (tracts in the 60-89th percentiles); and nonpoor (tracts below the 60th percentile). In Wave 1, L.A.FANS randomly selected and interviewed adults and children from over 3,000 households across the 65 sampled tracts [47]. In Wave 2, an attempt was made to re-interview all respondents in the original sample, while also interviewing a sample of newcomers to each neighborhood, but standard in-person interviews with health-related questions were only administered to those who remained in L.A. County [48]. The State University of New York at Buffalo Institutional Review Board approved all study protocols and the use of L.A.FANS restricted data. Consent was waived because L.A.FANS is a secondary data source.

Of the roughly 2,600 originally sampled adults (age 18 and over), 1,187 were interviewed in Wave 2. Once 34 respondents who did not report an activity space location are excluded, there are 1,153 panel respondents. Due to insufficient sample sizes of other ethnic groups, we limit our study to Latino, non-Latino White, and non-Latino Black adults, resulting in 1,065 respondents. An additional 19 respondents were removed for having missing data on any of the analysis variables, yielding a final sample of 1,046 adult respondents. With a negligible portion of the sample with missing data (1.8%), we employ listwise deletion, rather than multiple imputation. We structure our data longitudinally such that each observation represents one person-period, resulting in a total analytic sample of 2,092 person-periods.

L.A.FANS has respondent attrition between Waves 1 and 2. To address this issue, L.A.FANS provides panel weights to be used in all longitudinal analyses, which are a combination of the Wave 1 design weight and a Wave 2 attrition adjustment. Panel weights are designed to account for the oversampling of census tracts in the poorest strata of L.A. County, the oversampling of households with children, and the attrition of eligible Wave 1 panel members due to non-response [48]. These panel weights are also designed to make the sample representative of the L.A. County adult population at Wave 1 who reside in the county at Wave 2. L.A.FANS staff derived the attrition factor by executing logistic regression models using Wave 1 variables to predict non-response among panel respondents who at Wave 2 were not known to be ineligible (e.g., deceased, incarcerated). The inverse of the predicted probability of non-response obtained from the logistic regression models was used as the attrition weight [48]. A comparison of Wave 1 baseline characteristics indicates that panel respondents typically have more children, education, and income, as well as higher rates of employment and homeownership than respondents who left the panel.

L.A.FANS is an ideal source of data for studying how neighborhood exposures matter for individual health and well-being in Los Angeles [e.g., 42, 43, 49–55]. A key advantage of the L.A.FANS is the availability of census tract identifiers based on where respondents live, in addition to several locations respondents frequent and spend time (i.e., activity spaces). More specifically, L.A.FANS interviewers asked respondents to report the locations of five major activities: their current workplace (for all jobs), where they typically shop for groceries, where they worship, and where they obtain healthcare for illnesses and preventative care. Respondents were permitted to report up to three locations per activity in Wave 1 and up to four in Wave 2. For each activity location, respondents reported the addresses or cross-streets, from which geocodes were generated by L.A.FANS staff [48]. Another unique feature of L.A.FANS data is the ability to create neighborhood-level measures of social organization from survey items (described below). Incorporating the amount of time into our contextual exposures is a final novel benefit of using L.A.FANS.

Using this tract-level detail, we append census tract information from Census 2000 and the 2005–2009 American Community Survey to Waves 1 and 2 respondent-level data, respectively, and construct measures of adults’ neighborhood and activity space racial/ethnic and socioeconomic exposures. Despite the limitations of using census tracts as proxies for neighborhoods they are designed to be standardized in terms of their demographic, social, and economic characteristics, as well as being demarcated by discernible physical boundaries, such as bodies of water and bridges. All census tracts have been normalized to 2000 boundaries.

### Measures

#### Hypertension

The dependent variable is a dichotomous self-reported measure indicating whether the respondent has hypertension. Specifically, respondents were asked “Has a doctor ever told you that you have high blood pressure or hypertension?”

#### Neighborhood measures

We include two measures of neighborhood social organization. *Neighborhood organizational participation* captures whether the respondent participated in a local voluntary association during the past year across nine types of groups (e.g., neighborhood block meeting). Second, *collective efficacy* reflects residents’ perceptions of social cohesion and informal social control at the neighborhood level [27]. Social cohesion is measured with five L.A.FANS questions capturing whether respondents perceive their neighborhood as close-knit, trustworthy, helpful, friendly, and sharing common values. Informal social control is based on four survey questions addressing the likelihood that neighbors would intervene if children in the neighborhood were disrespecting adults, skipping school, or vandalizing property, and whether adults are watchful of the neighborhood. The specific survey questions that comprise neighborhood organizational participation and collective efficacy are presented in S1 Table.

To derive our neighborhood social organization measures, we follow a well-documented “ecometric” approach to creating aggregates of survey responses pertaining to respondents’ neighborhood perceptions and behaviors [19, 27, 36, 56]. To this end, we execute three-level item response models (items nested within individuals nested within census tracts) and use empirical Bayes estimates (EB residuals) to arrive at each neighborhood’s organizational participation and collective efficacy scores, the details of which have been described elsewhere [36].

There are two neighborhood structural measures: *socioeconomic disadvantage* and *co-ethnic density*. Socioeconomic disadvantage is a widely used composite measure of neighborhood SES [57] comprised of five variables (all percentages): individuals living below the poverty line, individuals in the labor force unemployed, households on public assistance, female-headed households with children, and individuals 25 and over who did not graduate from high school. Having neighbors of the same race/ethnicity may improve individual health through the diffusion of healthy behaviors and information [20]. Co-ethnic density is the percentage of the neighborhood population that matches the race/ethnicity as the respondent based on three groups: Latino, non-Latino White, and non-Latino Black.

We also use L.A.FANS data to estimate respondents’ average time per week spent in the following activity locations: workplace, grocery store, place of worship, and healthcare. Following prior work described elsewhere [54, 55], we derive exposure weights for each respondent and then apply these weights to their home and activity space measures to arrive at new exposure-weighted scores (e.g., neighborhood collective efficacy exposure). Activity space exposure measures represent a weighted average across all respondents’ activity space contexts that reflects individuals’ overall activity space exposures rather than separate measures for each activity location [54, 55]. The global activity space measure is preferred here because the separate activity space measures (e.g., workplace, grocery store) are highly correlated with one another, whereas correlations between residential neighborhood exposure measures and overall activity space exposure measures are weak to moderate. By weighting these contextual variables by exposure, they now reflect personal contextual exposure measures at the individual level [58]. Note that collective efficacy and organizational participation have only residential exposure versions because they are based on L.A.FANS survey questions pertaining to the respondent’s current neighborhood of residence, and sample sizes across activity space neighborhoods were insufficient to create activity space social organization measures.

#### Individual covariates

Beyond binary indicators for our three racial/ethnic groups (Latino, non-Latino Black, and non-Latino White), our models adjust for a range of individual-level covariates implicated in past research on neighborhoods and chronic conditions [e.g., 13, 17, 19, 55, 57]. Demographic characteristics include age (years) and binary indicators for whether the respondent is female, foreign born, married, and has children under 18 in the household. Socioeconomic characteristics are family income—the sum of earned and transfer household income in 2007 dollars and transformed using the inverse hyperbolic sine (IHS) function to account for zeros; education (years); and whether the respondent is employed and has health insurance. Additional individual-level controls are length of residence (IHS-transformed years lived in the current neighborhood) and survey wave. Table 1 presents survey-weighted descriptive statistics for all analysis variables.

**Table 1:** Weighted descriptive statistics for analysis variables for the total sample and racial/ethnic groups. Data from L.A.FANS Waves 1 and 2.

	Total Sample	Black	White	Latino
**Variables**	**Mean (SD) / %**	**Mean (SD) / %**	**Mean (SD) / %**	**Mean (SD) / %**
Self-reported hypertension	21.75	41.77	21.48	18.05
Race/ethnicity				
Black	9.10			
White	45.05			
Latino	45.85			
Residential neighborhood characteristics				
Organizational participation	0.34 (1.16)	0.32 (1.10)	0.64 (1.35)	0.04 (0.86)
Collective efficacy	0.30 (0.99)	0.20 (0.88)	0.63 (1.07)	0.01 (0.81)
Socioeconomic disadvantage	0.07 (0.78)	0.41 (0.88)	-0.41 (0.60)	0.48 (0.64)
Co-ethnic density	35.56 (21.32)	12.77 (10.54)	38.94 (20.52)	36.75 (21.00)
Activity space characteristics				
Socioeconomic disadvantage	-0.02 (0.17)	0.02 (0.17)	-0.06 (0.17)	0.02 (0.15)
Co-ethnic density	6.00 (7.22)	1.90 (2.90)	7.37 (8.09)	5.46 (6.50)
Individual-level covariates				
Age (years)	44.05 (15.52)	43.27 (16.20)	48.52 (16.15)	39.80 (13.39)
Female	48.00	59.86	43.67	49.89
Foreign born	40.84	3.55	11.31	77.24
Married	49.36	28.47	53.42	49.51
Presence of children	46.80	50.51	30.65	61.94
Family income (1000s)	60.77 (73.62)	52.52 (37.21)	83.05 (96.79)	40.52 (37.99)
Education (years)	13.29 (4.29)	14.52 (2.29)	15.75 (2.75)	10.62 (4.27)
Employed	69.77	63.48	69.39	71.38
Uninsured	25.75	11.79	13.05	40.99
Length of residence (years)	9.85 (10.59)	8.79 (8.97)	11.96 (12.38)	7.99 (8.38)
N (person-periods)	2,092	216	622	1,254

Note: L.A.FANS, Los Angeles Family and Neighborhood Survey; SD, standard deviation. Residential neighborhood and activity space measures are weighted by exposure. Family income is adjusted to 2007 dollars.

### Statistical analysis

To examine the study’s first objective, we estimate associations between neighborhood social organization exposure and hypertension risk by executing a series of random effects logistic models. We choose a random effects model because of the longitudinal and multilevel structure of the data. Recall that our neighborhood exposure measures are at the respondent level resulting in a two-level model—time (survey wave) nested within individuals. Here, we prefer the random effects model to the fixed effects model because of its ability to examine both time-invariant and time-varying variables, as well as our substantive interest in between-effects (i.e., racial/ethnic disparities) [59]. Our modeling strategy proceeds as follows: Model 1 presents the baseline racial/ethnic gap in hypertension risk, with Black adults as the reference group. Model 2 enters our neighborhood social organization measures (organizational participation and collective efficacy). Model 3 adjusts for Model 2 variables and includes residential socioeconomic disadvantage and co-ethnic density, while Model 4 controls for activity space versions of socioeconomic disadvantage and co-ethnic density. The full model (Model 5) adjusts for our individual-level controls. For ease of interpretation, we convert logistic regression coefficients to average marginal effects (AMEs) with 95% confidence intervals (CIs) derived from robust standard errors clustered at the individual level. We also report the intraclass correlation (ICC) for each model.

To address our second aim, we examine whether the hypertension effects of neighborhood social organization vary across racial/ethnic groups. Specifically, both neighborhood organizational participation and collective efficacy are interacted with race/ethnicity in separate fully adjusted random effects logistic models. The results are illustrated by plotting predicted probabilities of having hypertension by levels of neighborhood social organization with 95% CIs.

For our final objective, we explore sources of the racial/ethnic gaps in hypertension risk—between Black and White adults and Black and Latino adults—using Fairlie’s extension of the Blinder-Oaxaca decomposition technique for nonlinear models [60]. A common approach to assessing the contributing factors to racial/ethnic disparities in high blood pressure and other chronic diseases [61, 62], decomposition methods construct a counterfactual reflecting how the Black-White gap in hypertension would change, for example, if Black adults had the same neighborhood and individual characteristics as White adults. To do so, we use estimates from group-specific logistic models and partition Black-White (and Black-Latino) differences into the part explained by observed characteristics and an unexplained part, which reflects group differences in unobserved characteristics. See S1 Appendix for a detailed description of our application of the nonlinear decomposition of hypertension disparities. We apply L.A.FANS panel survey weights to all analyses, which were executed using Stata 16 [63].

## Results

### Multivariable model results

Table 2 presents results from a series of random effects logistic regression models predicting hypertension risk. Recall that our contextual exposure (residential and activity space) measures have been adjusted for the average amount of time adults spend per week in each context. In Model 1, we see that a large statistically significant disparity in the unadjusted probability of having hypertension exists between Black adults and both White and Latino adults. For example, White and Latino adults have a 19.0 and 20.5 percentage-point lower probability of having hypertension, respectively, than Black adults. Model 2 provides some evidence that living in neighborhoods with higher levels of organizational participation reduces hypertension risk (AME = -0.020, 95% CI = -0.044 to 0.004, *p* = 0.09), while collective efficacy does not reach statistical significance. Introducing residential social structural characteristics in model 3 considerably diminishes the Black-White hypertension gap (AME = -0.134, 95% CI = -0.217 to -0.052). In addition, residential socioeconomic disadvantage exposure is associated with a significantly higher probability of being hypertensive (AME = 0.051, 95% CI = 0.014 to 0.088).

**Table 2:** Average marginal effects estimated from random effects logistic models predicting adult hypertension risk. Data from L.A.FANS Waves 1 and 2.

Variables	Model 1	Model 2	Model 3	Model 4	Model 5
Race/ethnicity (ref = Black)					
White	-.190***	-.175***	-.134***	-.125**	-.135***
	(-.260, -.120)	(-.247, -.103)	(-.217, -.052)	(-.210, -.041)	(-.210, -.061)
Latino	-.205***	-.209***	-.201***	-.195***	-.145***
	(-.273, -.137)	(-.277, -.141)	(-.273, -.130)	(-.268, -.123)	(-.221, -.069)
Residential neighborhood characteristics					
Organizational participation		-.020	-.016	-.018	-.027*
		(-.044, .004)	(-.040, .008)	(-.042, .006)	(-.050, -.004)
Collective efficacy		-.009	.003	.002	-.003
		(-.035, .016)	(-.026, .032)	(-.027, .031)	(-.031, .025)
Socioeconomic disadvantage			.051**	.050**	.049**
			(.014, .088)	(.012, .087)	(.013, .085)
Co-ethnic density			-.001	.000	-.010
			(-.011, .010)	(-.011, .010)	(-.022, .001)
Activity space characteristics					
Socioeconomic disadvantage				-.038	-.044
				(-.190, .115)	(-.195, .107)
Co-ethnic density				-.029	-.009
				(-.066, .008)	(-.050, .032)
ICC	0.674	0.678	0.682	0.678	0.589
Individual controls	No	No	No	No	Yes

Note: *N* = 2,092 person-periods. L.A.FANS, Los Angeles Family and Neighborhood Survey; ICC, intra-class correlation. 95% confidence intervals in parentheses. Individual controls include age, gender, nativity, marital status, presence of children, family income, education, employment status, insurance status, and length of neighborhood residence. All models include survey wave. All residential neighborhood and activity space measures are weighted by exposure.

* p < .05;

** p < .01;

*** p < .001.

Model 4 of Table 2 adjusts for activity space socioeconomic disadvantage and co-ethnic density exposures. Doing so slightly reduces the magnitude of the Black-White and Black-Latino gaps in hypertension, and these activity space social structural exposures are not significantly associated with hypertension risk. In the fully adjusted model (Model 5), neighborhood organizational participation is significantly associated with a lower probability of having high blood pressure (AME = -.027, CI = -.050 to -.004). We also see that accounting for the complete battery of contextual and individual characteristics reduces the Black-White and Black-Latino hypertension disparities to 13.5 and 14.5 percentage points, respectively, but the gaps remain statistically significant (see S2 Table for full model results).

### Do the effects of neighborhood social organization vary across racial/ethnic groups?

**Fig 1:**
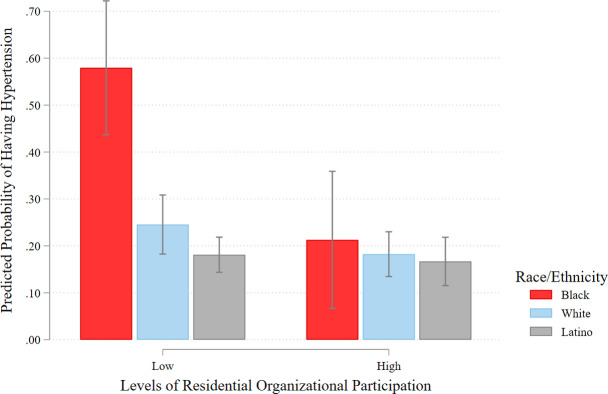
Predicted probabilities of having hypertension by levels of neighborhood organizational participation and racial/ethnic group. Data from L.A.FANS Waves 1 and 2. Estimates from fully adjusted random effects logistic model.

We also examine whether the hypertension effects of exposure to neighborhood social organizational characteristics vary across Black, Latino, and White adults. To do so, we interact our neighborhood organizational participation and collective efficacy measures with race/ethnicity in separate fully adjusted random effects logistic models—i.e., including all the variables from Model 5 in Table 2 (for interaction model results, see Models 6a and 6b in S2 Table). In Fig 1, we present predicted probabilities and 95% CIs of hypertension risk based on high (one standard deviation above the mean) and low (one standard deviation below the mean) levels of residential organizational participation for Black, White, and Latino adults. The figure illustrates a strong protective effect for Black individuals living in neighborhoods where their neighbors are actively involved in local organizations and associations. For example, the probability of being hypertensive is over 63% lower when Black adults reside in neighborhoods with high (compared with low) levels of organizational participation (.58 vs. .21, *p* < .05). By contrast, neighborhood participation plays a minimal role on hypertension risk among White and Latino adults. Also noteworthy, the racial gap in high blood pressure is effectively eliminated at high levels of organizational participation, as evidenced by the overlapping confidence intervals.

**Fig 2:**
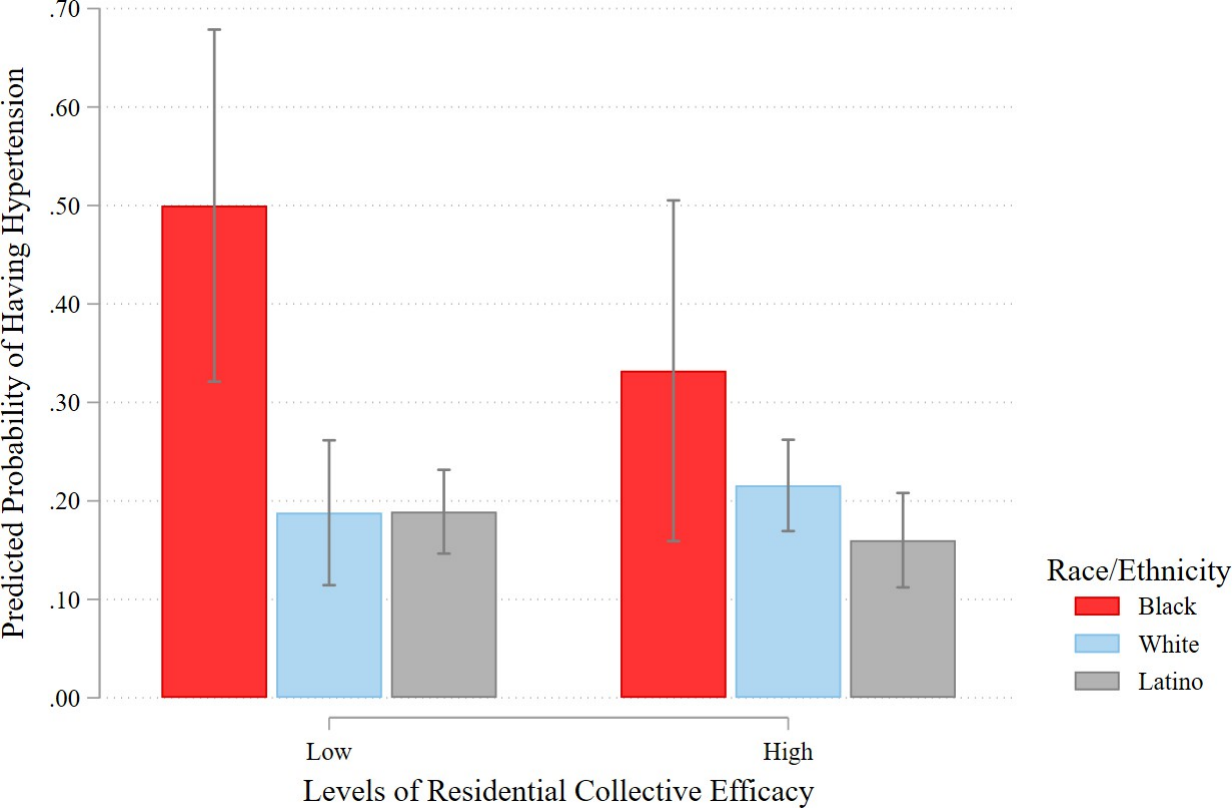
Predicted probabilities of having hypertension by levels of neighborhood collective efficacy and racial/ethnic group. Data from L.A.FANS Waves 1 and 2. Estimates from fully adjusted random effects logistic model.

In Fig 2, a similar pattern exists where the Black disadvantage in hypertension is significant at low levels of neighborhood collective efficacy, whereas the Black-White and Black-Latino gaps are not significantly different when considering highly efficacious residential neighborhoods. Though imprecise, there is some evidence that the probability that Black adults have high blood pressure is reduced by 34% when living in high (versus low) collective efficacy neighborhoods (.50 vs. .33, *p* = n.s.).

### Decomposition of racial/ethnic disparities in hypertension risk

**Fig 3:**
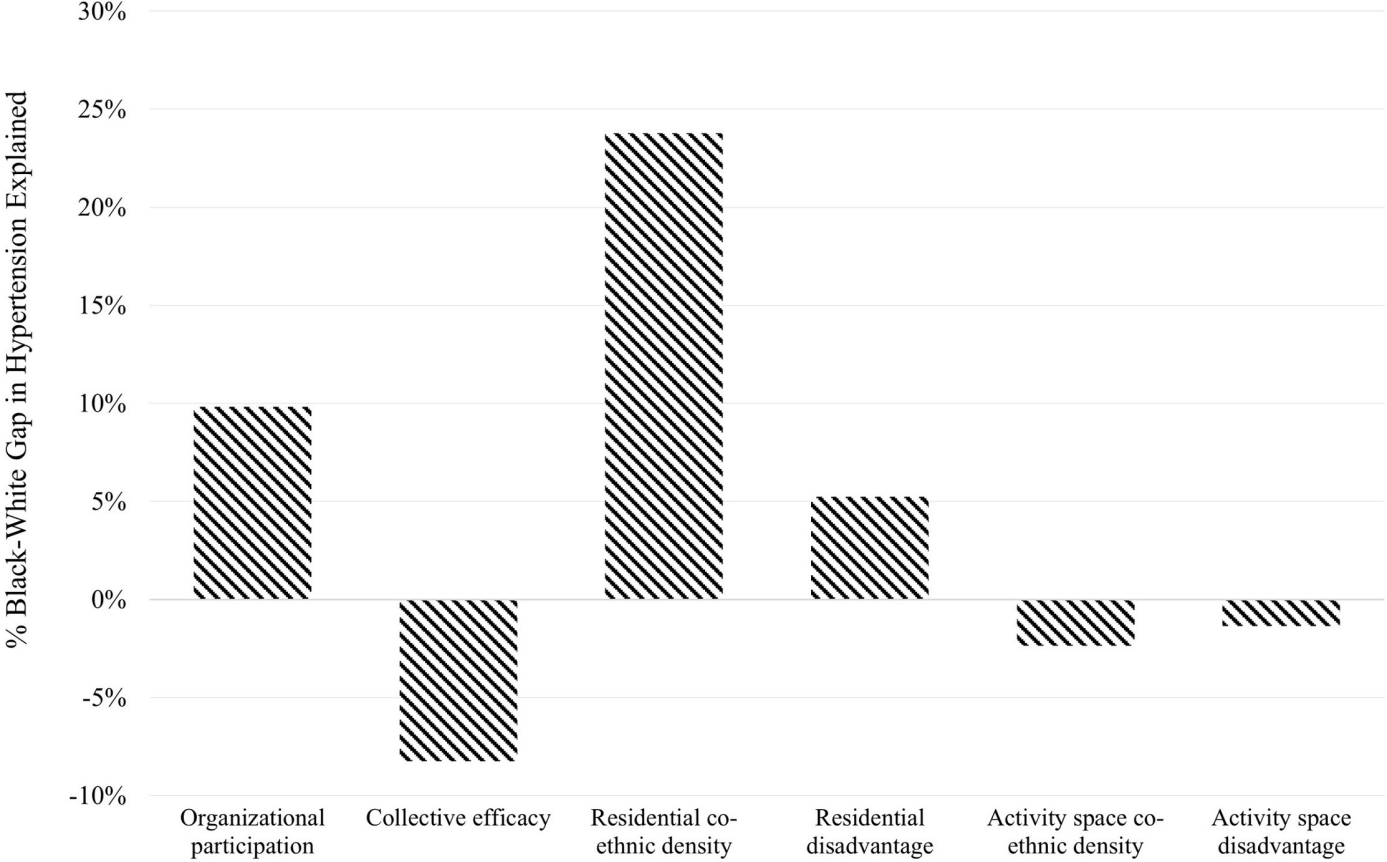
Decomposition of Black-White differences in hypertension risk by neighborhood exposure characteristics. Whites are used as the reference group. The y-axis represents the percentage of the hypertension gap explained by each variable or group of variables.

Next, we employ a counterfactual nonlinear decomposition [60] to examine how racial/ethnic disparities in hypertension risk would change if Black adults had the same neighborhood social environment characteristics, as well as individual characteristics, as White and Latino adults, respectively. In Fig 3, each bar represents the percentage of the Black-White gap (20.3 percentage points, see Table 1) explained by social environment characteristics. Beginning with neighborhood social organizational features, almost 10% of the Black-White hypertension disparity is due to different levels of residential organizational participation in Black and White adults’ home neighborhoods. By contrast, the negative percentage explained for neighborhood collective efficacy indicates that the Black-White gap would widen by 8.2% if Black and White residents lived in neighborhoods with comparable levels of collective efficacy. Fig 3 also shows that racial differences in levels of residential co-ethnic density explain nearly 25% of the Black-White hypertension gap. An additional 5.3% of the gap is explained by Black-White disparities in levels of residential socioeconomic disadvantage. Finally, equalizing Black and White levels of activity space social structural environments would increase the hypertension disparity by roughly 4%.

**Fig 4:**
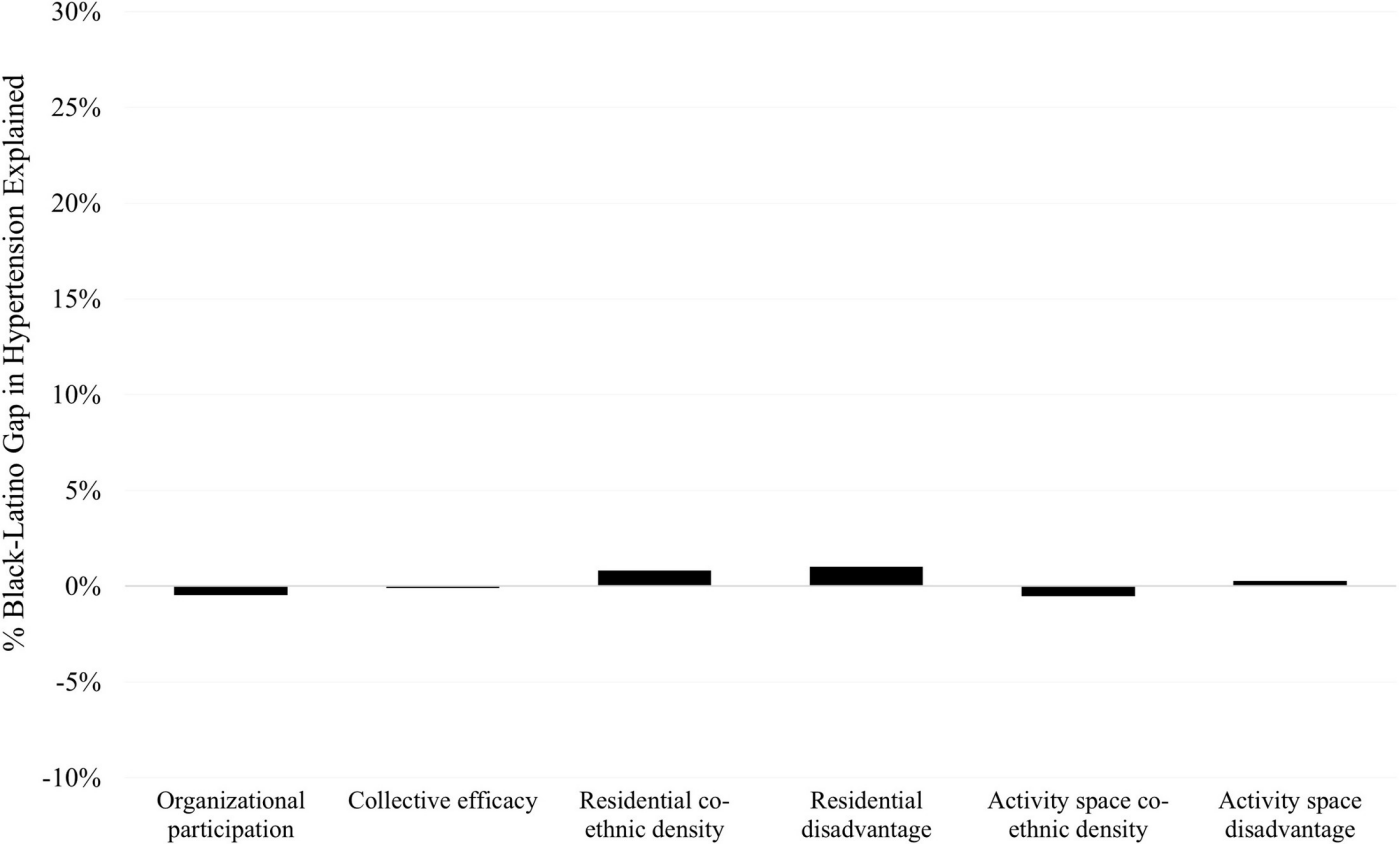
Decomposition of Black-Latino differences in hypertension risk by neighborhood exposure characteristics. Latinos are used as the reference group. The y-axis represents the percentage of the hypertension gap explained by each variable or group of variables.

With respect to the observed Black-Latino hypertension disparity, Fig 4 illustrates that a minimal portion of the Black-Latino hypertension gap can be attributed to neighborhood social organizational and structural characteristics. Our observed variables are not substantial sources of the Black-Latino gap, which is unsurprising considering that in Los Angeles, Black and Latino adults face comparable risk factors in terms of their place exposures and individual characteristics (see Table 1).

## Discussion

Drawing on recent advances in place and health effects research and analyzing unique multilevel data on Los Angeles County residents, we investigate whether and how neighborhood social organizational characteristics matter for hypertension risk and contribute to racial/ethnic hypertension disparities. Our findings reveal that neighborhood organizational participation is associated with a lower probability of being hypertensive. This suggests that living in neighborhoods where people are involved in informal and formal organizations and associations (e.g., neighborhood watch, civic groups, ethnic pride organizations) may protect residents against excessive exposure to area stressors that can elevate the risks of developing high blood pressure.

Comparatively, we find that neighborhood collective efficacy (social cohesion, expectations for informal social control) is not significantly associated with being hypertensive. This is consistent with one study reporting a null effect of neighborhood social cohesion on hypertension [18], but contrary to another study finding that social cohesion is associated with a lower risk of hypertension [19].

More compelling, we find that the protective effect of neighborhood organizational participation is significantly stronger for Black adults than Latino and White adults, respectively. Black residents who live in highly organized communities have a lower risk of being hypertensive by over 60% compared with living in neighborhoods with low levels of organizational involvement where the Black disadvantage in hypertension risk is at its widest. Moreover, at high levels of neighborhood organizational participation, these observed hypertension differences between Black adults and Latino and White adults are substantially narrowed and no longer statistically significant (see Fig 1). This is perhaps unsurprising, given that prior research on neighborhood collective action in Los Angeles indicates that Black residents organize, affiliate, and mobilize to solve local neighborhood problems (e.g., crime, disorder), thereby reducing stress and blood pressure levels [35]. Though not as striking as organizational participation, we also show that the hypertension effects of neighborhood collective efficacy vary across racial/ethnic groups, such that the hypertension disparity between Black adults and Latino and White adults is no longer significant at high levels of collective efficacy. Echoing extant work, this highlights the salience of neighborhood social connectedness, mutual trust, and a willingness to act on behalf of fellow neighbors as a potential stress-buffering mechanism for African Americans [32].

Results from our decomposition of the racial/ethnic gap in hypertension shows that almost one-fifth of the Black-White hypertension disparity can be explained by differences in the neighborhood social organization exposures of Black and White adults’ residential communities. In addition, almost a quarter of the Black-White gap in high blood pressure can be attributed to differential exposures to neighborhood co-ethnic density, which is in line with studies reporting protective ethnic density effects for racial/ethnic minority health outcomes and behaviors [20, 64]. To better understand how neighborhood social exposures contribute to hypertension risk among Black and Latino adults, neighborhood organizational participation and other stress-buffering mechanisms should be prioritized in future research. One ethnographic study of Black adults with hypertension, for example, points to excessive contextual stressors, such as unsafe local surroundings and a lack of access to health-promoting resources (adequate healthcare, healthy food options) as exacerbating high blood pressure [65]. Another potential mechanism we encourage researchers to investigate is the evolving built environment in Los Angeles, particularly the role of community organizations, such as nonprofits [66], libraries [67], and other routine organizations geared toward improving the health, safety, and overall well-being of low-income, communities of color [68]. Gentrification processes should also be explored, as recent work suggests that living in gentrifying neighborhoods is equally beneficial for Black, White, and Latino residents [69, 70].

Aligning with the burgeoning activity space and health literature, we consider both spatial and temporal dimensions of exposure by the amount of time adults spend in their residential and nonresidential neighborhoods. Doing so provides more conservative estimates of neighborhood effects on hypertension risk and avoids common pitfalls associated with conventional neighborhood studies that do not account for individuals’ daily mobility over time and space [71, 72]. Chaix and colleagues [45], for example, refer to this type of bias as the “residential effect fallacy,” which results from not accounting for individuals’ nonresidential exposures and the subsequent confounding with residential exposures. This is corroborated in other studies noting that people’s daily mobility exposures to nonresidential places confounds or attenuates residential neighborhood effects on health [46, 50]. In our study, we find that our exposure-weighted contextual results are substantively similar to unweighted results, but we consider these estimates more conservative than those using measures based on the residential neighborhood not accounting for durations of exposure.

Our study has some limitations. Due to our use of L.A.FANS data, our results may not be generalizable beyond Los Angeles County. In addition, our dependent variable is based on a self-reported measure of hypertension, rather than resting seated blood pressure measurements. Prior research has shown that individuals may not be aware that they have hypertension, and while self-reports may underreport the prevalence of high blood pressure, there is general consistency with physician diagnoses [73–75]. Yet, our prevalence estimates for Black, Latino, and White adults are in line with both self-reported and measured hypertension from nationally representative surveys [76–78]. Even more important, our survey-weighted results are on par with measured hypertension prevalence estimates from Los Angeles County during our study timeframe [7]. Thus, we consider any underreporting of hypertension in the L.A.FANS to be minimal and not induce substantial bias into our estimates, and that our conclusions regarding the role of neighborhood social organization exposures and hypertension risk in Los Angeles would hold for measured hypertension.

Another limitation is that L.A.FANS does not ascertain an exhaustive list of respondents’ routine activities and their locations. If some routine activities occur outside the residential neighborhood that are not captured by L.A.FANS (e.g., visits with family and friends) then exposure weights for our activity space measures will be underestimated and our contextual estimates biased toward zero. Linking individual-focused epidemiological data to creative, theoretically grounded area-level measures should be a top priority in future research. These include use of GPS tracking [79], ecological momentary assessments (EMAs) [80], and qualitative interviews to better capture spatial and temporal dynamics of adult neighborhood exposures. On this front, researchers should pursue spatially fluid indicators of community social organization that can add insights into how different ethnic groups engage with and perceive their home and away contexts. Finally, given that our neighborhood social exposure measures are based on residential compositions, these may or may not be indicative of the actual social environments to which people are exposed at different times of the day. The racial/ethnic and socioeconomic daily trajectories of neighborhoods may evolve throughout the day, and thus real-time estimates of exposure should be collected to gain more precise neighborhood exposure effects on health behaviors and outcomes.

In conclusion, that racial/ethnic disparities in hypertension persist even when accounting for multiple neighborhood and individual factors suggests that researchers are presented with challenges for thinking about the myriad mechanisms through which social and behavioral conditions impact biological states and conditions. A logical starting point for conceptualization and measurement is longstanding structural racism, which, in the U.S., has profoundly shaped the residential and broader geographical circumstances of not only Black Americans, but immigrants and other people of color [81, 82]. Extending this more broadly to international contexts (for which research on contextual determinants of blood pressure has predominantly focused on residential neighborhoods versus activity spaces), future research must consider the potential impacts of urban and rural policies that shape the daily health-related circumstances, opportunities, and risks of residential neighborhoods as well as the locations that different populations occupy or inhabit throughout their daily routines.

## Supporting information

S1 Table Survey items that comprise neighborhood social organization measures, L.A.FANS. (PDF)

S2 Table Logit coefficients from the full random effects logistic model and interaction models, L.A.FANS. (PDF)

S1 Appendix Description of nonlinear decomposition of racial/ethnic hypertension disparities. (DOCX)

## References

[1] Agyemang C, Kunst A, Bhopal R, Zaninotto P, Unwin N, Nazroo J, et al. A cross-national comparative study of blood pressure and hypertension between English and Dutch South-Asian–and African-origin populations: The role of national context. Am J Hypertens. 2010;23(6):639–48. doi: 10.1038/ajh.2010.39 20300070

[2] Tsao CW, Aday AW, Almarzooq ZI, Alonso A, Beaton AZ, Bittencourt MS, et al. Heart disease and stroke statistics—2022 update: A report from the American Heart Association. Circulation. 2022;145(8). doi: 10.1161/CIR.0000000000001052 35078371

[3] Aggarwal R, Chiu N, Wadhera RK, Moran AE, Raber I, Shen C, et al. Racial/ethnic disparities in hypertension prevalence, awareness, treatment, and control in the United States, 2013 to 2018. Hypertension. 2021;78(6):1719–26. doi: 10.1161/HYPERTENSIONAHA.121.17570 34365809 PMC10861176

[4] Hardy ST, Chen L, Cherrington AL, Moise N, Jaeger BC, Foti K, et al. Racial and ethnic differences in blood pressure among US adults, 1999–2018. Hypertension. 2021;78(6):1730–41. doi: 10.1161/HYPERTENSIONAHA.121.18086 34719937 PMC8851685

[5] Thomas SJ, Booth JN, Dai C, Li X, Allen N, Calhoun D, et al. Cumulative incidence of hypertension by 55 years of age in Blacks and Whites: The CARDIA study. J Am Heart Assoc. 2018;7(14):e007988. 29997132 10.1161/JAHA.117.007988 PMC6064834

[6] Eidem E, Nagano S, Steinberg L, Johnson E, Lightstone AS, Cui Y, et al. Los Angeles County Department of Public Health Office of Women’s Health. 2017 p. 28.

[7] Hales CM, Carroll MD, Simon PA, Kuo T, Ogden CL. Hypertension prevalence, awareness, treatment, and control among adults aged ≥18 years—Los Angeles County, 1999–2006 and 2007–2014. MMWR Morb Mortal Wkly Rep. 2017;66(32):846–9. 28817553 10.15585/mmwr.mm6632a3 PMC5657669

[8] Bassett DR, Fitzhugh EC, Crespo CJ, King GA, McLaughlin JE. Physical activity and ethnic differences in hypertension prevalence in the United States. Prev Med. 2002;34(2):179–86. doi: 10.1006/pmed.2001.0969 11817913

[9] Williams DR, Mohammed SA, Leavell J, Collins C. Race, socioeconomic status and health: Complexities, ongoing challenges and research opportunities. Ann N Y Acad Sci. 2010;1186:69–101. doi: 10.1111/j.1749-6632.2009.05339.x 20201869 PMC3442603

[10] Cuevas AG, Williams DR, Albert MA. Psychosocial factors and hypertension. Cardiol Clin. 2017;35(2):223–30. 28411896 10.1016/j.ccl.2016.12.004 PMC5407387

[11] Brondolo E, Love EE, Pencille M, Schoenthaler A, Ogedegbe G. Racism and hypertension: A review of the empirical evidence and implications for clinical practice. Am J Hypertens. 2011;24(5):518–29. doi: 10.1038/ajh.2011.9 21331054

[12] Hicken MT, Lee H, Morenoff J, House JS, Williams DR. Racial/ethnic disparities in hypertension prevalence: Reconsidering the role of chronic stress. Am J Public Health. 2014;104(1):117–23. doi: 10.2105/AJPH.2013.301395 24228644 PMC3910029

[13] Claudel SE, Adu-Brimpong J, Banks A, Ayers C, Albert MA, Das SR, et al. Association between neighborhood-level socioeconomic deprivation and incident hypertension: A longitudinal analysis of data from the Dallas heart study. Am Heart J. 2018;204:109–18. doi: 10.1016/j.ahj.2018.07.005 30092412 PMC6217793

[14] Cubbin C, Hadden WC, Winkleby MA. Neighborhood context and cardiovascular disease risk factors: The contribution of material deprivation. Ethn Dis. 2001;11(4):687–700. 11763293

[15] Wagner KJP, Boing AF, Subramanian S, Höfelmann DA, D’Orsi E. Effects of neighborhood socioeconomic status on blood pressure in older adults. Rev Saúde Pública. 2016;50:78. doi: 10.1590/S1518-8787.2016050006595 28099662 PMC5152802

[16] Kershaw KN, Robinson WR, Gordon-Larsen P, Hicken MT, Goff DC, Carnethon MR, et al. Association of changes in neighborhood-level racial residential segregation with changes in blood pressure among black adults: The CARDIA study. JAMA Intern Med. 2017;177(7):996. doi: 10.1001/jamainternmed.2017.1226 28505341 PMC5710452

[17] Gao X, Kershaw KN, Barber S, Schreiner PJ, Do DP, Diez Roux AV, et al. Associations between residential segregation and incident hypertension: The Multi‐Ethnic Study of Atherosclerosis. J Am Heart Assoc. 2022;11(3):e023084. doi: 10.1161/JAHA.121.023084 35048712 PMC9238487

[18] Kaiser P, Diez Roux AV, Mujahid M, Carnethon M, Bertoni A, Adar SD, et al. Neighborhood environments and incident hypertension in the Multi-Ethnic Study of Atherosclerosis. Am J Epidemiol. 2016;183(11):988–97. doi: 10.1093/aje/kwv296 27188946 PMC4887578

[19] Mujahid MS, Diez Roux AV, Morenoff JD, Raghunathan TE, Cooper RS, Ni H, et al. Neighborhood characteristics and hypertension. Epidemiology. 2008;19(4):590–8. doi: 10.1097/EDE.0b013e3181772cb2 18480733

[20] Bécares L, Shaw R, Nazroo J, Stafford M, Albor C, Atkin K, et al. Ethnic density effects on physical morbidity, mortality, and health behaviors: A systematic review of the literature. Am J Public Health. 2012;102(12):e33–66. doi: 10.2105/AJPH.2012.300832 23078507 PMC3519331

[21] Viruell-Fuentes EA, Ponce NA, Alegría M. Neighborhood context and hypertension outcomes among Latinos in Chicago. J Immigr Minor Health. 2012;14(6):959–67. doi: 10.1007/s10903-012-9608-4 22527740

[22] Agyemang C, van Hooijdonk C, Wendel-Vos W, Ujcic-Voortman JK, Lindeman E, Stronks K, et al. Ethnic differences in the effect of environmental stressors on blood pressure and hypertension in the Netherlands. BMC Public Health. 2007;7:118. doi: 10.1186/1471-2458-7-118 17587458 PMC1919368

[23] Mujahid MS, Roux AVD, Cooper RC, Shea S, Williams DR. Neighborhood stressors and race/ethnic differences in hypertension prevalence (The Multi-Ethnic Study of Atherosclerosis). Am J Hypertens. 2011;24(2):187–93. doi: 10.1038/ajh.2010.200 20847728 PMC3319083

[24] Mujahid MS, Moore LV, Petito LC, Kershaw KN, Watson K, Diez Roux AV. Neighborhoods and racial/ethnic differences in ideal cardiovascular health (the Multi-Ethnic Study of Atherosclerosis). Health Place. 2017;44:61–9. doi: 10.1016/j.healthplace.2017.01.005 28167269 PMC5354104

[25] Mayne SL, Moore KA, Powell-Wiley TM, Evenson KR, Block R, Kershaw KN. Longitudinal associations of neighborhood crime and perceived safety with blood pressure: The Multi-Ethnic Study of Atherosclerosis (MESA). Am J Hypertens. 2018;31(9):1024–32. doi: 10.1093/ajh/hpy066 29897398 PMC6077783

[26] Kershaw KN, Diez Roux AV, Burgard SA, Lisabeth LD, Mujahid MS, Schulz AJ. Metropolitan-level racial residential segregation and black-white disparities in hypertension. Am J Epidemiol. 2011;174(5):537–45. doi: 10.1093/aje/kwr116 21697256 PMC3202148

[27] Sampson RJ, Raudenbush SW, Earls F. Neighborhoods and violent crime: A multilevel study of collective efficacy. Science. 1997;277(5328):918–24. doi: 10.1126/science.277.5328.918 9252316

[28] Browning CR, Cagney KA. Neighborhood structural disadvantage, collective efficacy, and self-rated physical health in an urban setting. J Health Soc Behav. 2002;43(4):383–99. 12664672

[29] Cohen DA, Finch BK, Bower A, Sastry N. Collective efficacy and obesity: The potential influence of social factors on health. Soc Sci Med. 2006;62(3):769–78. doi: 10.1016/j.socscimed.2005.06.033 16039767

[30] Ullmann SH, Goldman N, Pebley AR. Contextual factors and weight change over time: A comparison between U.S. Hispanics and other population sub-groups. Soc Sci Med. 2013;90:40–8. doi: 10.1016/j.socscimed.2013.04.024 23746607 PMC4533836

[31] Cagney KA, Browning CR. Exploring neighborhood-level variation in asthma and other respiratory diseases: The contribution of neighborhood social context. J Gen Intern Med. 2004;19(3):229–36. doi: 10.1111/j.1525-1497.2004.30359.x 15009777 PMC1492148

[32] Hughes-Halbert C, Bellamy S, Briggs V, Bowman M, Delmoor E, Kumanyika S, et al. Collective efficacy and obesity-related health behaviors in a community sample of African Americans. J Community Health. 2014;39(1):124–31. doi: 10.1007/s10900-013-9748-z 24026302 PMC4017594

[33] Jackson N, Denny S, Sheridan J, Zhao J, Ameratunga S. The role of neighborhood disadvantage, physical disorder, and collective efficacy in adolescent alcohol use: A multilevel path analysis. Health Place. 2016;41:24–33. doi: 10.1016/j.healthplace.2016.07.005 27521816

[34] Ahern J, Galea S. Collective efficacy and major depression in urban neighborhoods. Am J Epidemiol. 2011;173(12):1453–62. doi: 10.1093/aje/kwr030 21527512 PMC3145397

[35] Altschuler A, Somkin CP, Adler NE. Local services and amenities, neighborhood social capital, and health. Soc Sci Med. 2004;59(6):1219–29. doi: 10.1016/j.socscimed.2004.01.008 15210093

[36] Carpiano RM. Neighborhood social capital and adult health: An empirical test of a Bourdieu-based model. Health Place. 2007;13(3):639–55. doi: 10.1016/j.healthplace.2006.09.001 17084655

[37] Stockdale SE, Wells KB, Tang L, Belin TR, Zhang L, Sherbourne CD. The importance of social context: neighborhood stressors, stress-buffering mechanisms, and alcohol, drug, and mental health disorders. Soc Sci Med. 2007;65(9):1867–81. doi: 10.1016/j.socscimed.2007.05.045 17614176 PMC2151971

[38] Swaroop S, Morenoff JD. Building community: The neighborhood context of social organization. Soc Forces. 2006;84(3):1665–95.

[39] Al-Bayan M, Islam N, Edwards S, Duncan DT. Neighborhood perceptions and hypertension among low-income black women: A qualitative study. BMC Public Health. 2016;16(1):1075. doi: 10.1186/s12889-016-3741-2 27733142 PMC5062878

[40] Coulon SM, Wilson DK, Alia KA, Van Horn ML. Multilevel associations of neighborhood poverty, crime, and satisfaction with blood pressure in African-American adults. Am J Hypertens. 2016;29(1):90–5. doi: 10.1093/ajh/hpv060 25917562 PMC5014129

[41] Cagney KA, York Cornwell E, Goldman AW, Cai L. Urban mobility and activity space. Annu Rev Sociol. 2020;46(1):623–48.

[42] Browning CR, Calder CA, Krivo LJ, Smith AL, Boettner B. Socioeconomic segregation of activity spaces in urban neighborhoods: Does shared residence mean shared routines? RSF Russell Sage Found J Soc Sci. 2017;3(2):210–31. doi: 10.7758/RSF.2017.3.2.09 29034322 PMC5640327

[43] Krivo LJ, Washington HM, Peterson RD, Browning CR, Calder CA, Kwan MP. Social isolation of disadvantage and advantage: The reproduction of inequality in urban space. Soc Forces. 2013;92(1):141–64.

[44] Shareck M, Frohlich KL, Kestens Y. Considering daily mobility for a more comprehensive understanding of contextual effects on social inequalities in health: A conceptual proposal. Health Place. 2014;29:154–60. doi: 10.1016/j.healthplace.2014.07.007 25103785

[45] Chaix B, Duncan D, Vallée J, Vernez-Moudon A, Benmarhnia T, Kestens Y. The “residential” effect fallacy in neighborhood and health studies: Frmal definition, empirical identification, and correction. Epidemiology. 2017;28(6):789–97. 28767516 10.1097/EDE.0000000000000726

[46] Kwan MP. The neighborhood effect averaging problem (neap): An elusive confounder of the neighborhood effect. Int J Environ Res Public Health. 2018;15(9):1841. doi: 10.3390/ijerph15091841 30150510 PMC6163400

[47] Peterson CE, Sastry N, Pebley AR, Ghosh-Dastidar B, Williamson S, Lara-Cinisomo S. The Los Angeles Family and Neighborhood Survey: Codebook. RAND Corporation; 2004.

[48] Peterson CE, Pebley AR, Sastry N, Yuhas K, Ghosh-Dastidar B, Haas AC, et al. The Los Angeles Family and Neighborhood Survey, Wave 2: User’s Guide and Codebook. RAND Corporation; 2011.

[49] Inagami S, Cohen DA, Finch BK. Non-residential neighborhood exposures suppress neighborhood effects on self-rated health. Soc Sci Med. 2007 Oct 1;65(8):1779–91. doi: 10.1016/j.socscimed.2007.05.051 17614175

[50] Sharp G, Denney JT, Kimbro RT. Multiple contexts of exposure: Activity spaces, residential neighborhoods, and self-rated health. Soc Sci Med. 2015;146:204–13. doi: 10.1016/j.socscimed.2015.10.040 26519605

[51] Browning CR, Calder CA, Boettner B, Tarrence J, Khan K, Soller B, et al. Neighborhoods, activity spaces, and the span of adolescent exposures. Am Sociol Rev. 2021;86(2):201–33. doi: 10.1177/0003122421994219 34992302 PMC8725782

[52] Browning CR, Calder CA, Soller B, Jackson AL, Dirlam J. Ecological networks and neighborhood social organization. Am J Sociol. 2017;122(6):1939–88. doi: 10.1086/691261 29379218 PMC5786432

[53] Jones M, Pebley AR. Redefining neighborhoods using common destinations: Social characteristics of activity spaces and home census tracts compared. Demography. 2014;51(3):727–52. doi: 10.1007/s13524-014-0283-z 24719273 PMC4048777

[54] Kimbro RT, Sharp G, Denney JT. Home and away: Area socioeconomic disadvantage and obesity risk. Health Place. 2017 Mar 1;44:94–102. 10.1016/j.healthplace.2017.02.001 28214705

[55] Sharp G, Kimbro RT. Neighborhood social environments, healthy resources, and adult diabetes: Accounting for activity space exposures. Health Place. 2021;67:102473. doi: 10.1016/j.healthplace.2020.102473 33212395

[56] Mujahid MS, Diez Roux AV, Morenoff JD, Raghunathan T. Assessing the measurement properties of neighborhood scales: From psychometrics to ecometrics. Am J Epidemiol. 2007;165(8):858–67. doi: 10.1093/aje/kwm040 17329713

[57] Morenoff JD, House JS, Hansen BB, Williams DR, Kaplan GA, Hunte HE. Understanding social disparities in hypertension prevalence, awareness, treatment, and control: The role of neighborhood context. Soc Sci Med. 2007;65(9):1853–66. doi: 10.1016/j.socscimed.2007.05.038 17640788 PMC2705439

[58] Kwan MP. From place-based to people-based exposure measures. Soc Sci Med. 2009;69(9):1311–3. doi: 10.1016/j.socscimed.2009.07.013 19665828

[59] Bell A, Fairbrother M, Jones K. Fixed and random effects models: Making an informed choice. Qual Quant. 2019;53(2):1051–74.

[60] Fairlie RW. An extension of the Blinder-Oaxaca decomposition technique to logit and probit models. J Econ Soc Meas. 2005;30(4):305–16.

[61] Basu S, Hong A, Siddiqi A. Using decomposition analysis to identify modifiable racial disparities in the distribution of blood pressure in the United States. Am J Epidemiol. 2015;182(4):345–53. doi: 10.1093/aje/kwv079 26199379 PMC4528957

[62] Gaskin DJ, Zare H, Jackson JW, Ibe C, Slocum J. Decomposing race and ethnic differences in CVD risk factors for mid-life women. J Racial Ethn Health Disparities. 2021;8(1):174–85. doi: 10.1007/s40615-020-00769-9 32462612

[63] StataCorp. Stata Statistical Software: Release 16. College Station, TX: StataCorp LLC; 2019.

[64] Yang TC, Lei L, Kurtulus A. Neighborhood ethnic density and self-rated health: Investigating the mechanisms through social capital and health behaviors. Health Place. 2018;53:193–202. doi: 10.1016/j.healthplace.2018.08.011 30172823 PMC6172945

[65] Koehler K, Lewis L, F. Cronholm P. Neighborhood and social influences on blood pressure: An exploration of causation in the explanatory models of hypertension among African Americans. J Community Med. 2018;1:1002.

[66] Sharkey P, Torrats-Espinosa G, Takyar D. Community and the crime decline: The causal effect of local nonprofits on violent crime. Am Sociol Rev. 2017;82(6):1214–40.

[67] Klinenberg E. Palaces for the People: How Social Infrastructure Can Help Fight Inequality, Polarization, and the Decline of Civic Life. New York: Penguin Random House LLC; 2018.

[68] Small ML, Gose LE. How do low-income people form survival networks? Routine organizations as brokers. Ann Am Acad Pol Soc Sci. 2020;689(1):89–109.

[69] Agbai CO. Shifting neighborhoods, shifting health: A longitudinal analysis of gentrification and health in Los Angeles County. Soc Sci Res. 2021;100:102603. doi: 10.1016/j.ssresearch.2021.102603 34627559 PMC8505760

[70] Smith GS, McCleary RR, Thorpe RJ. Racial disparities in hypertension prevalence within US gentrifying neighborhoods. Int J Environ Res Public Health. 2020;17(21):7889. doi: 10.3390/ijerph17217889 33126467 PMC7662342

[71] Matthews SA, Yang TC. Spatial polygamy and contextual exposures (spaces): Promoting activity space approaches in research on place and health. Am Behav Sci. 2013;57(8):1057–81. doi: 10.1177/0002764213487345 24707055 PMC3975622

[72] Kwan MP. The uncertain geographic context problem. Ann Assoc Am Geogr. 2012;102(5):958–68.

[73] Wellman JL, Holmes B, Hill SY. Accuracy of self‐reported hypertension: Effect of age, gender, and history of alcohol dependence. J Clin Hypertens. 2020;22(5):842–9. doi: 10.1111/jch.13854 32277600 PMC8029970

[74] Yoon SSS, Ostchega Y, Louis T. Recent trends in the prevalence of high blood pressure and its treatment and control, 1999–2008. NCHS Data Brief. 2010 Oct;(48):1–8. 21050532

[75] Brown AF, Ang A, Pebley AR. The relationship between neighborhood characteristics and self-rated health for adults with chronic conditions. Am J Public Health. 2007;97(5):926–32. doi: 10.2105/AJPH.2005.069443 17395847 PMC1854885

[76] Borrell LN, Crawford ND. Disparities in self-reported hypertension in Hispanic subgroups, non-Hispanic black and non-Hispanic white adults: The National Health Interview Survey. Ann Epidemiol. 2008;18(10):803–12. doi: 10.1016/j.annepidem.2008.07.008 18922396 PMC2604123

[77] Fang J, Yang Q, Ayala C, Loustalot F. Disparities in access to care among US adults with self-reported hypertension. Am J Hypertens. 2014;27(11):1377–86. doi: 10.1093/ajh/hpu061 24847953 PMC4263941

[78] Ong KL, Cheung BMY, Man YB, Lau CP, Lam KSL. Prevalence, awareness, treatment, and control of hypertension among United States adults 1999–2004. Hypertension. 2007;49(1):69–75. doi: 10.1161/01.HYP.0000252676.46043.18 17159087

[79] Zenk SN, Schulz AJ, Matthews SA, Odoms-Young A, Wilbur J, Wegrzyn L, et al. Activity space environment and dietary and physical activity behaviors: A pilot study. Health Place. 2011;17(5):1150–61. doi: 10.1016/j.healthplace.2011.05.001 21696995 PMC3224849

[80] York Cornwell E, Goldman AW. Neighborhood disorder and distress in real time: Evidence from a smartphone-based study of older adults. J Health Soc Behav. 2020;61(4):523–41. doi: 10.1177/0022146520967660 33210544

[81] Hicken MT, Kravitz-Wirtz N, Durkee M, Jackson JS. Racial inequalities in health: Framing future research. Soc Sci Med. 2018;199:11–8. doi: 10.1016/j.socscimed.2017.12.027 29325781 PMC5915332

[82] Williams DR, Lawrence JA, Davis BA. Racism and health: Evidence and needed research. Annu Rev Public Health. 2019;40(1):105–25. doi: 10.1146/annurev-publhealth-040218-043750 30601726 PMC6532402

