# Supplementary material for: Neighborhood social organization exposures and racial/ethnic disparities in hypertension risk in Los Angeles
Source: PLoS One. 2023 Mar 6;18(3):e0282648. doi: 10.1371/journal.pone.0282648 (PMC9987829; doi:10.1371/journal.pone.0282648)
Supplement: S1 Table — (PDF) [file pone.0282648.s001.pdf]

S1 Table. Survey items that comprise neighborhood social organization measures, L.A.FANS.

Measure and Survey Questions	Response Range
Collective efficacy	
Social cohesion	(1) strongly agree — (5) strongly disagree
1. "This is a close-knit neighborhood."*	
2. "People in this neighborhood can be trusted."*	
3. "People in this neighborhood do not share the same values."	
4. "People around here are willing to help their neighbors."*	
5. "People in this neighborhood generally do not get along with each other."	
Informal social control	
1. "If a group of neighborhood children were skipping school and hanging out on a street corner, how likely is it that neighbors would do something about it?"*	(1) very likely — (5) very unlikely
2. "If some children were spray-painting graffiti on a local building, how likely is it that neighbors would do something about it?"*	(1) very likely — (5) very unlikely
3. "If a child was showing disrespect to an adult, how likely is it that people in your neighborhood would scold that child?"*	(1) very likely — (5) very unlikely
4. "You can count on adults in this neighborhood to watch out that children are safe and do not get in trouble."*	(1) strongly agree — (5) strongly disagree
Organizational participation	
"In the past 12 months, have you yourself participated in the following activities?"	(0) no — (1) yes
1. Neighborhood or block organization meeting	
2. Business or civic group (e.g., Masons, Elks, Rotary Club)	
3. Nationality or ethnic pride club	
4. A local or state political organization	
5. Volunteered in a local organization	
6. Veterans' group	
7. Labor union	
8. Literary, art, study, or discussion group	
9. Fraternity, sorority, or alumni group	

Note: *Indicates reverse-coded. L.A.FANS, Los Angeles Family and Neighborhoods Survey.
