# Supplementary material for: Neighborhood social organization exposures and racial/ethnic disparities in hypertension risk in Los Angeles
Source: PLoS One. 2023 Mar 6;18(3):e0282648. doi: 10.1371/journal.pone.0282648 (PMC9987829; doi:10.1371/journal.pone.0282648)
Supplement: S2 Table — (PDF) [file pone.0282648.s002.pdf]

S2 Table. Logit coefficients from the full random effects logistic model and interaction models, L.A.FANS.

Variables	Model 5	Model 6a	Model 6b
Race/ethnicity (ref = Black)			
White	-1.66***<br>(-2.62, -0.70)	-1.87***<br>(-2.93, -0.81)	-1.78***<br>(-2.80, -0.77)
Latino	-1.73***<br>(-2.68, -0.78)	-1.95***<br>(-2.98, -0.91)	-1.73***<br>(-2.71, -0.75)
Residential neighborhood characteristics			
Organizational participation	-0.32*<br>(-0.59, -0.04)	-1.28*<br>(-2.30, -0.25)	-0.33*<br>(-0.60, -0.07)
Collective efficacy	-0.03<br>(-0.36, 0.30)	-0.03<br>(-0.36, 0.30)	-0.61<br>(-1.76, 0.55)
Race/ethnicity X neighborhood social organization interaction (ref = Black)			
White X organization participation		0.99<br>(-0.08, 2.07)	
Latino X organization particpation		1.21*<br>(0.13, 2.28)	
White X collective efficacy			0.75<br>(-0.48, 1.97)
Latino X collective efficacy			0.45<br>(-0.74, 1.63)
Socioeconomic disadvantage	0.58**<br>(0.14, 1.02)	0.58**<br>(0.13, 1.03)	0.53*<br>(0.08, 0.98)
Co-ethnic density	-0.12<br>(-0.27, 0.02)	-0.15*<br>(-0.28, -0.01)	-0.15*<br>(-0.29, -0.01)
Activity space characteristics			
Socioeconomic disadvantage	-0.53<br>(-2.32, 1.27)	-0.51<br>(-2.36, 1.35)	-0.50<br>(-2.28, 1.28)
Co-ethnic density	-0.11<br>(-0.60, 0.38)	-0.07<br>(-0.54, 0.39)	-0.09<br>(-0.56, 0.39)
Individual-level covariates			

Age	0.10***<br>(0.08, 0.13)	0.10***<br>(0.08, 0.13)	0.10***<br>(0.08, 0.13)
Female	0.52<br>(-0.03, 1.06)	0.50<br>(-0.06, 1.06)	0.49<br>(-0.06, 1.04)
Foreign-born	-0.44<br>(-1.10, 0.23)	-0.43<br>(-1.11, 0.25)	-0.43<br>(-1.10, 0.24)
Married	0.53<br>(-0.03, 1.09)	0.53<br>(-0.05, 1.11)	0.55<br>(-0.02, 1.12)
Presence of children	0.22<br>(-0.43, 0.86)	0.25<br>(-0.41, 0.92)	0.23<br>(-0.42, 0.87)
Family income (IHS-transformed)	-0.02<br>(-0.16, 0.12)	-0.02<br>(-0.16, 0.12)	-0.02<br>(-0.16, 0.12)
Education (years)	-0.03<br>(-0.11, 0.04)	-0.03<br>(-0.11, 0.04)	-0.03<br>(-0.11, 0.04)
Employed	-0.27<br>(-1.07, 0.53)	-0.40<br>(-1.06, 0.27)	-0.32<br>(-1.04, 0.40)
Uninsured	0.23<br>(-0.46, 0.92)	0.31<br>(-0.39, 1.01)	0.26<br>(-0.44, 0.95)
Legnth of residence (IHS-transformed)	0.14<br>(-0.13, 0.41)	0.14<br>(-0.13, 0.41)	0.14<br>(-0.13, 0.41)
Survey wave	0.40<br>(-0.15, 0.95)	0.44<br>(-0.11, 0.99)	0.44<br>(-0.10, 0.98)
ICC	0.589	0.587	0.587

Note: N = 2,092 person-periods. L.A.FANS, Los Angeles Family and Neighborhood Survey; ICC, intra-class correlation; IHS, inverse hyperbolic sine. 95% confidence intervals in parentheses. All residential neighborhood and activity space measures are weighted by exposure.

* p < .05;

** p < .01;

*** p < .001.
