# Supplementary material for: Neighborhood social organization exposures and racial/ethnic disparities in hypertension risk in Los Angeles
Source: PLoS One. 2023 Mar 6;18(3):e0282648. doi: 10.1371/journal.pone.0282648 (PMC9987829; doi:10.1371/journal.pone.0282648)
Supplement: S1 Appendix — (DOCX) [file pone.0282648.s003.docx]

**S1 Appendix. Description of nonlinear decomposition of racial/ethnic hypertension disparities.**

The Fairlie decomposition method employs estimates from our logistic models and partitions Black-White (or Black-Latino) differences into the part explained by observed characteristics (the compositional or endowment effect), and an unexplained part, which reflects group differences in unobserved characteristics (the coefficient effect). Using Black and White adults as an illustration, we express the nonlinear decomposition model as:

$$\bar{Y}^{W}- \bar{Y}^{B}= \left[ \sum_{i=1}^{N^{W}} \frac{\Phi\left( \mathbf{X}_{i}^{W}\hat{\beta}^{W} \right)}{N^{W}} - \sum_{i=1}^{N^{B}} \frac{\Phi\left( \mathbf{X}_{i}^{B}\hat{\beta}^{W} \right)}{N^{B}} \right] + \left[ \sum_{i=1}^{N^{B}} \frac{\Phi\left( \mathbf{X}_{i}^{B}\hat{\beta}^{W} \right)}{N^{B}} - \sum_{i=1}^{N^{B}} \frac{\Phi\left( \mathbf{X}_{i}^{B}\hat{\beta}^{B} \right)}{N^{B}} \right] , (1)$$

where $\bar{Y}^{j}$ represents that average probability of having hypertension for adults with race *j* (for White adults *W* and Black adults *B*), $\mathbf{X}^{j}$ is a vector of independent variables, *N* is the sample size of each group, $\hat{\beta}$ represents a vector of coefficients from the logistic regression equation *Y* = $\Phi\left( \mathbf{X}\hat{\beta} \right)$, where $\Phi\left( . \right)$ is the cumulative distribution function of the logistic distribution of *Y*. The first term on the right-hand side of equation 1 refers to the part of the racial gap in hypertension risk attributed to compositional differences, weighted by the coefficients for White adults. The second term corresponds to the part of the disparity due to differences in coefficients, or the differences in Black-White processes in determining hypertension risk [60]. We use the coefficients for White adults as the reference because of our interest in the comparison—that is, we are interested in understanding how the racial gap in hypertension would change if Black adults had the same contextual and individual characteristics as White adults.

Interpreting the unexplained portion of the gap is challenging, which directs our interest on the first term of equation 1 (i.e., the explained part). Thus, the contribution of each variable to the Black-White gap in hypertension risk is equal to the change in the average predicted probability of having hypertension when assigning Black respondents the distribution of White respondents for each variable, holding the remaining variables’ distributions constant. The independent contribution of a covariate $\mathbf{X}_{1}$ is expressed in equation 2:

$$\frac{1}{N^{B}} \sum_{i=1}^{N^{B}} \Phi\left( \hat{\alpha}^{*}+ \mathbf{X}_{1i}^{W}\hat{\beta}_{1}^{*}+ \mathbf{X}_{2i}^{W}\hat{\beta}_{2}^{*} \right) - \Phi\left( \hat{\alpha}^{*}+ \mathbf{X}_{1i}^{B}\hat{\beta}_{1}^{*}+ \mathbf{X}_{2i}^{W}\hat{\beta}_{2}^{*} \right), (2)$$

where $\hat{\beta}^{*}$ represents logistic coefficients from the pooled sample, which address the violation of the assumption that the White and Black sample sizes are comparable [60]. To circumvent this assumption, we randomly draw a subsample of the larger group, Whites, to equal the smaller group, Blacks, and then average our decomposition results over 1,000 subsamples. Because results can be sensitive to the ordering of the independent variables, we randomize the order for each iteration.
